#  An Exploratory Investigation of Some Statistical Summaries of Oximeter Oxygen Saturation Data from Preterm Babies

**DOI:** 10.5402/2011/296418

**Published:** 2011-04-26

**Authors:** Dominic S. Lee, Marina Zahari, Glynn Russell, Brian A. Darlow, Carl J. Scarrott, Marco Reale

**Affiliations:** ^1^Department of Mathematics and Statistics, University of Canterbury, Christchurch 8140, New Zealand; ^2^Imperial College Healthcare NHS Trust, London W2 1NY, UK; ^3^Department of Paediatrics, Christchurch School of Medicine and Health Sciences, University of Otago, Christchurch 8140, New Zealand

## Abstract

*Aim*. To explore the potential usefulness of the mean, standard deviation (SD), and coefficient of variation (CV = SD/mean) of oximeter oxygen saturations in the clinical care of preterm babies. *Methods*. This was an exploratory investigation involving 31 preterm babies at 36 weeks postmenstrual age. All babies were healthy, but two were considered to be clinically unstable and required greater attention. Each baby's oxygen saturations were recorded using an oximeter and summarized by the mean, SD, and CV. The potential usefulness of each measure was assessed by its ability to distinguish the two unstable babies from the others. This was achieved using box plots and hierarchical clustering together with the Calinski-Harabasz (CH) index to quantify clustering performance (higher CH index indicates stronger clustering outcome). *Results*. The box plots flagged both unstable babies as outliers and none of the other babies. Successful clustering of the stable and unstable babies was achieved using the CV (CH = 72.8) and SD (CH = 63.3) but not with the mean. *Conclusion*. Taking the box plots and clustering results together, it seems plausible that variability might be more effective than mean level for detecting instability in oxygen saturation in preterm babies and that the combination of variability and level through the CV might be even better.

## 1. Introduction

Preterm babies display physiological instabilities such as low oxygen saturation level and variations of heart rate and respiratory patterns [1]. These instabilities arise from a combination of level of arousal, immature organ development, neonatal illnesses [2], clinical interventions [3], and environmental factors (temperature, light, and noise). This makes it difficult to distinguish instabilities that are merely due to prematurity from those partly caused by neonatal illnesses that affect cardiorespiratory functions. 

Fetal heart rate variability has been quantified to monitor fetal well-being in high-risk pregnancies and labour [4, 5]. Validated measures of prediction of neonatal illness have been developed based on heart rate characteristics [6, 7]. Indices or scores have been calculated from respiratory support data to describe the severity of pulmonary status and respiratory failure in ventilated preterm babies [8, 9]. However, a similar level of understanding has not been reached for oxygen saturation. It is known that an inappropriate amount of oxygen can be detrimental to premature babies—with both hypoxaemia and hyperoxaemia capable of tissue injury [10]—but what the appropriate amount is and what features of oxygen saturation are useful are still unclear [11].

A convenient source of oxygen saturation measurements is the pulse oximeter, which is commonly used in many modern neonatal intensive care units. This investigation was prompted by the availability of a set of oximeter oxygen saturation data from 31 preterm babies. Due to the small sample size, the goal of the investigation had to be kept modest, seeking to explore the potential usefulness of some common statistical summaries of the data. Since oxygen saturation level has been of considerable interest [12–19], one of the statistics investigated is the mean. Another feature of oxygen saturation that has received some attention is variability [12, 20–22]; hence, the second statistic chosen is the standard deviation (SD). The third is the coefficient of variation (CV), which is the ratio of the SD to the mean, thus providing a composite measure of variability relative to the mean level.

## 2. Materials and Methods

### 2.1. The Study Cohort and Data

The oxygen saturation data that were used in this investigation had been collected for a separate study approved by the Upper South B Regional Ethics Committee in New Zealand. Babies were eligible for that study if they had been admitted to the neonatal intensive care unit at Christchurch Women's Hospital between November 2004 and January 2006 with gestation less than 36 weeks, had never received mechanical ventilation, or had required continuous positive airways pressure for less than twelve hours. Babies were also excluded if they had required treatment with opiates or sedative drugs, were born after maternal drug use in pregnancy, had known congenital malformations involving the cardiorespiratory system, or had conditions requiring major surgery. At the time of entry to the study babies had to be clinically well, had oxygen saturation consistently greater than 92% and not requiring supplemental oxygen, and had not been treated with methylxanthine therapy in the previous seven days. The study depended on the availability of a Masimo Radical-7 pulse oximeter (Masimo Corporation, Irvine, Calif, USA) and research nurse time; hence not all eligible babies were enrolled. 

After parental consent was obtained, an enrolled baby was connected to the pulse oximeter and data acquisition commenced once a good waveform was attained. Data were collected for two groups of babies: (1) Group P, comprising 6 babies, each with one recording episode per week from 34 weeks postmenstrual age (PMA) until discharge and (2) Group N, consisting of 25 babies, each with only one recording episode at 36 weeks PMA. Each recording episode lasted for a continuous duration of at least 6 hours during which oxygen saturation readings were made once every two seconds. The subset of Group P's data taken at 36 weeks PMA was combined with the data for Group N to form the data set (from 31 babies altogether) used in this investigation. A summary of some demographic information for these 31 babies is given in Table 1.

Although all babies had to be clinically well to be eligible for study, two babies were identified as being clinically mildly unstable. Baby P17 was unstable at 34–36 weeks PMA. The day following enrolment at 34 weeks PMA, she had several episodes of apnea requiring stimulation and was placed on low-flow nasal oxygen, which was continued for 3 days. There was no evidence of sepsis or other problems. At 35 weeks PMA, she had oxygen saturations less than 90% for 24% of the time and desaturation episodes associated with feeds. She was again placed on low-flow nasal oxygen, which was continued for 48 hours. At 36 weeks PMA, she had brief desaturations with feeds and the low-flow nasal oxygen was recommenced for further 3 days. Baby N21 was unstable at 36 weeks PMA when oxygen saturations less than 90% were recorded for almost 30% of the monitoring period. Clinical review revealed no problems bar a soft systolic murmur and an echo-cardiogram was undertaken, which was normal but showed a small patent foramen ovale. The oxygen saturations were consistently 93% or greater over the next few days.

### 2.2. Statistical Methods

The Masimo Radical oximeter used was designed for maximum sensitivity for oxygen saturations between 70–100%. Readings below 70% were less reliable but as a new generation motion-tolerant low-perfusion oximeter [23], the Masimo Radical reduced errors from these causes. Suspect readings that occurred from unclear causes were retained by introducing a weighting function to down-weight them. This weighted each measurement between 70–100% by 1, while measurements between 0–69% were linearly weighted between 0 and 1. All subsequent analyses were based on the weighted measurements. These down-weighted measurements constituted a very small part (about 1.5%) of the total measurements that were recorded.

Each baby's oxygen saturation recordings were summarized using three statistical measures, the mean for measuring saturation level, SD for measuring saturation variability, and CV (=SD/mean) as a composite measure of variability relative to the mean level. The potential usefulness of each measure was assessed by its ability to distinguish the two unstable babies from the others. This was done in two ways. First, a qualitative graphical assessment was made using box plots. The common convention of labeling points that were more than 1.5 times the interquartile range (IQR) beyond the first or third quartile as outliers was adopted. The actual number of times the IQR that an outlier fell beyond the first or third quartile was used to indicate how extreme that outlier was and will be referred to as the extremeness index.

Next, a quantitative appraisal was performed by using each measure in turn to group the babies into clusters, such that babies within a cluster looked more “similar” with respect to the measure than babies from different clusters. This was done using the hierarchical clustering [24] method with squared Euclidean distance for determining the degree of similarity. Hierarchical clustering works by grouping unlabelled objects into homogeneous groups in an attempt to maximise the within-group similarity and minimise the between-group similarity at the same time. The extent to which this is achieved can be measured by an index of clustering performance, such as the Calinski-Harabasz (CH) [25] index. This index was found to be the best among 30 indices examined in Milligan and Cooper [26]. A higher value of the CH index serves as an indication of better separation between clusters and greater homogeneity within each cluster, hence a stronger clustering result overall. All computations were performed using the Matlab software package.

## 3. Results

Table 2 provides key summary statistics for all of the oxygen saturation measurements taken at 36 weeks PMA pooled from all 31 babies. The mean, SD, and CV of oxygen saturation for individual babies are given in Table 3.

### 3.1. Box Plots

**Figure 1:**
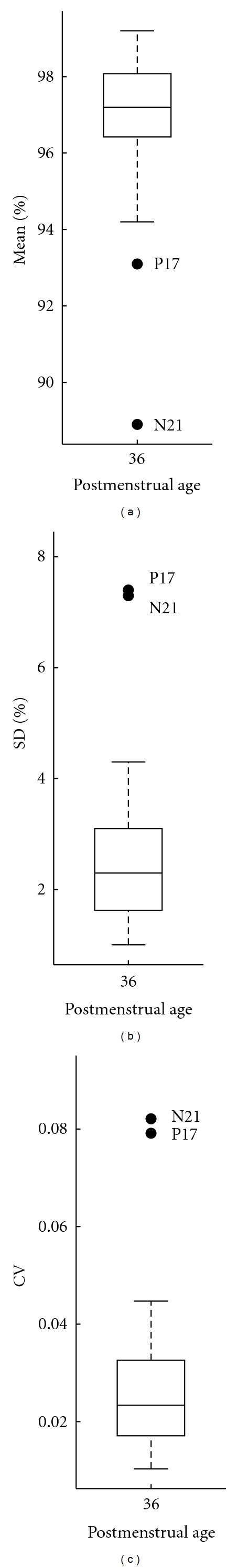
Box plots for the mean (a), SD (b), and CV (c).

The box plots for the means, SDs, and CVs of the 31 babies are shown in Figure 1.

Recall that, in a box plot, the lower and upper edges of the box denote the locations of the first and third quartiles, respectively. The line within the box denotes the location of the median. The difference between the third and first quartiles, which corresponds to the length of the box, is the IQR. The lower tail of the box plot extends from the lower edge to the smallest point that is within 1.5 times the IQR below the lower edge. Likewise, the upper tail extends from the upper edge of the box to the largest point that is within 1.5 times the IQR above the upper edge. Points that are more than 1.5 times the IQR beyond the edges of the box are regarded as outliers. Thus, the box plot for each statistical measure represents the empirical distribution of that measure across the 31 babies. The line within the box plot for the mean denotes the location of the median of the means and is situated at 97.2%; likewise, the median of the SDs is at 2.0% and the median of the CVs is at 0.023. Each box plot flags babies P17 and N21 as outliers. None of the other babies is flagged as an outlier. The values of the extremeness index for babies P17 and N21, respectively, are 2.02 and 4.56 with the mean, 2.92 and 2.85 with the SD, and 2.98 and 3.18 with the CV.

### 3.2. Clustering

**Figure 2:**
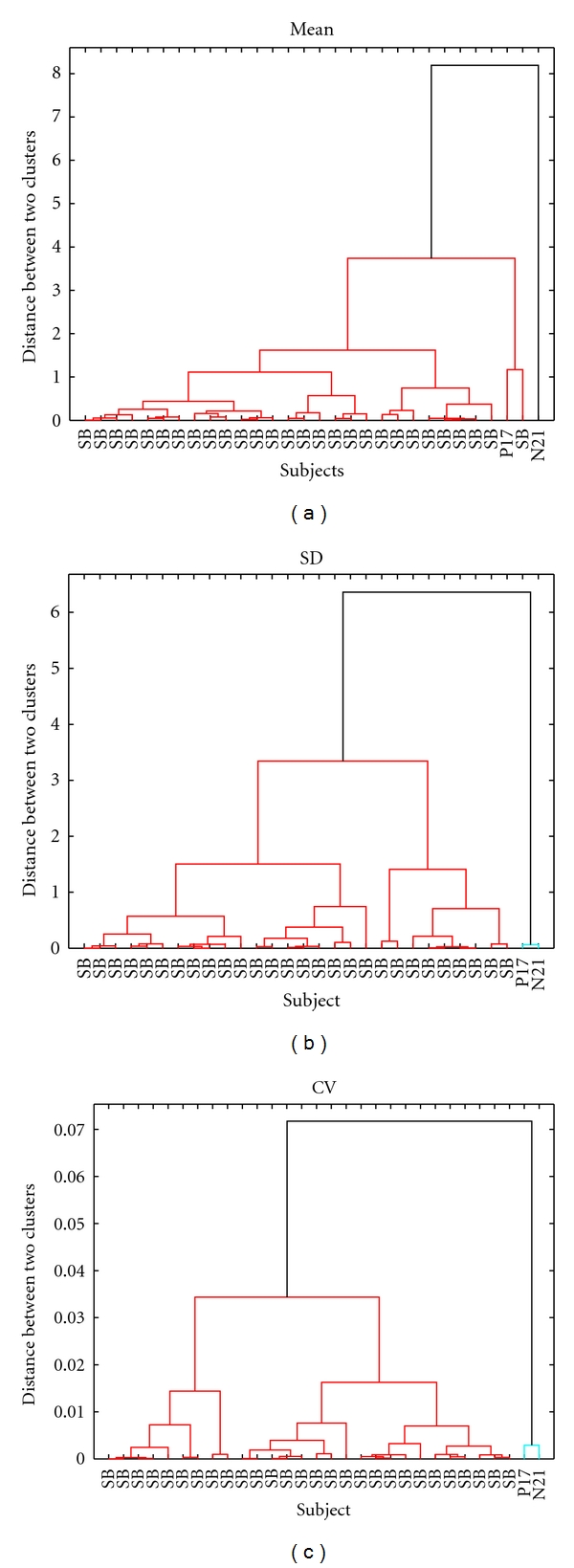
Dendrograms for hierarchical clustering with the mean (a), SD (b), and CV (c).

The results from hierarchical clustering using the mean, SD, and CV in turn are illustrated by dendrograms in Figure 2. A dendrogram shows possible clusters of objects labeled on the horizontal axis, with distances between clusters represented by the lengths of the vertical lines. To avoid cluttering the figure, babies P17 and N21 have been labeled explicitly but all of the other stable babies have simply been labeled as SB. By choosing which horizontal lines to cut, different number of clusters and clustering outcomes can emerge. The best choice is the one that maximises within-group similarity while minimising between-group similarity at the same time. In the figure, the best choice in each dendrogram is depicted using a common colour for objects belonging to the same group. For clustering with the mean, for example, the first dendrogram shows the best outcome as two groups, with N21 by itself in one group but with P17 together with all of the stable babies in the other group (coloured red). Thus, successful clustering of the stable and unstable babies into two groups is achieved using the CV (CH = 72.8) and SD (CH = 63.3) but not with the mean. Even though the clustering outcomes for CV and SD are the same—P17 and N21 in one group and all other stable babies in another group—the higher CH index for CV indicates that its clusters are better separated and more homogeneous within.

## 4. Discussion

Although not directly comparable to previous studies, which examined babies at different age ranges and pooled data from different ages, our data from 31 babies for 36 weeks PMA are still broadly similar in terms of oxygen saturation level. For example, for babies pooled from 29 to 36 weeks GA [18] and from 30 to 34 weeks GA [19] and studied between 2 to 14 days after birth, the medians of the means for oxygen saturation were 96.25% and 97%, respectively. From the box plot for the mean in Figure 1, the median of the means of the 31 babies at 36 weeks PMA is 97.2%. Other studies had included preterm babies with respiratory disorders [2–12], babies monitored within a laboratory setting [16], babies studied at 37 and 43 weeks PMA [17], and babies followed up at three-month intervals over the first 12-month postdelivery period [18]. Median or median baseline oxygen saturation levels ranging from 96.25% to 100% were obtained in these studies. From Table 2, the mean of our pooled data is 96.9% and the median is 98%. Studies involving full-term newborns found the median or median baseline of oxygen saturation to be 98.3% [13] in a 24-hour period from admission to discharge, about 98% in the first month of age [14] and between 98% and 100% in the 2 to 6 months after birth [14, 15].

The relatively low extremeness index of 2.02 for P17 exacerbated by the large value of 4.56 for N21 helps explain the unsuccessful clustering with the mean—P17 looks more like it belongs in a group with the stable babies than with N21 in an unstable group. With the SD or CV, the values of the extremeness index for P17 and N21 are close together and relatively large (around 3), thus aiding their clustering into a group apart from the other babies. The larger value of the CH index for the CV suggests that the resulting clusters are more pronounced than for the SD. This appears to be consistent with the larger values of the extremeness index for both P17 and N21 obtained from the CV box plot as compared to those from the SD. It therefore seems plausible that variability might be more effective than mean level for detecting instability in oxygen saturation in preterm babies and that the combination of variability and level through the CV might be even better.

Most previous studies on oxygen saturation in preterm babies concentrated on saturation level (mean, median, median of the means or median baseline) [12–19]. The results here add to the few known studies, such as that by DiPietro et al. [12], suggesting that variability may reflect perinatal health conditions even though a baby may appear to be well. The box plots in Figure 1 show that the use of mean level or variability (SD) separately can lead to conflicting views about which of the two unstable babies is at greater risk. The mean appears to suggest that N21 is much more unstable than P17 while the SD seems to show that P17 is slightly more unstable than N21. One way to resolve this is to use an appropriate composite measure combining mean level and variability, such as the CV.

The use of the CV for quantifying oxygen saturation in preterm babies is uncommon but not new. Saito et al. [20] and York et al. [21] used the CV to assess the effect of oxygen fluctuation on retinopathy of prematurity (ROP). They found that the CV of oxygen saturation was higher for babies with threshold ROP [21] or with stage 3 or higher ROP [20] within the first two weeks after birth. They concluded that increased fluctuation of arterial oxygen tension might increase the risk of developing threshold ROP in very-low-birth-weight premature babies. In another study examining the impact of echocardiography on the cardiorespiratory stability of preterm babies, Groves et al. [22] reported that the CV of blood pressure, heart rate, and oxygen saturation, albeit not clinically significant, were statistically higher during the echocardiography period than during the rest period.

The data set that was used was an opportune one for this investigation but its small sample size of 31, including only two unstable babies, was an obvious drawback that hampered stronger results. Nevertheless, the results do help to draw attention to the use of both mean level and viability in the continuing quest to better understand oxygen saturation in preterm babies.

## Figures and Tables

**Table 1:** Summary of gestational age (GA), birth weight, and gender.

GA in weeks mean (range)	Birth weight in g mean (range)	Gender male : female
32.9 (29–35)	2008 (1310–2810)	12 : 19

**Table 2:** Summary of oxygen saturation (%) data taken at 36 weeks PMA pooled from all 31 babies.

Mean (SD)	Median (IQR*)	5th, 95th percentiles
96.9 (3.6)	98 (96–99)	90, 100

*IQR expressed as lower quartile to upper quartile.

**Table 3:** CV, SD, and mean of oxygen saturation for individual babies at 36 weeks PMA.

Case no.	CV	SD	mean
P02	0.014	1.4	99.2
P03	0.023	2.3	98.2
P06	0.045	4.3	97.2
P10	0.014	1.4	97.7
P11	0.014	1.4	97.4
P17	0.079	7.4	93.1
N01	0.030	2.9	96.9
N02	0.032	3.2	97.1
N04	0.021	2.1	98.9
N05	0.020	1.9	99.0
N06	0.022	2.2	98.2
N13	0.038	3.6	97.1
N16	0.033	3.1	95.8
N17	0.032	3.1	96.4
N19	0.024	2.3	98.2
N21	0.082	7.3	88.9
N24	0.022	2.1	98.2
N26	0.033	3.1	96.0
N28	0.027	2.5	94.2
N36	0.044	4.2	96.5
N37	0.037	3.6	95.8
N52	0.024	2.3	97.6
N56	0.010	1.0	97.2
N57	0.020	2.0	96.8
N58	0.020	1.9	96.4
N66	0.016	1.5	97.4
N76	0.015	1.5	98.6
N77	0.017	1.6	97.4
N79	0.018	1.7	97.4
N81	0.024	2.4	97.5
N82	0.016	1.5	96.9
